# Ultra-Deep Sequencing of HIV-1 near Full-Length and Partial Proviral Genomes Reveals High Genetic Diversity among Brazilian Blood Donors

**DOI:** 10.1371/journal.pone.0152499

**Published:** 2016-03-31

**Authors:** Rodrigo Pessôa, Paula Loureiro, Maria Esther Lopes, Anna B. F. Carneiro-Proietti, Ester C Sabino, Michael P. Busch, Sabri S Sanabani

**Affiliations:** 1 Clinical Laboratory, Department of Pathology, LIM 03, Hospital das Clínicas (HC), School of Medicine, University of São Paulo, São Paulo, Brazil; 2 Pernambuco State Center of Hematology and Hemotherapy—HEMOPE, Recife, Pernambuco, Brazil; 3 Hemorio, Rio de Janeiro, Brazil; 4 Minas Gerais State Center of Hematology and Hemotherapy-HEMOMINAS, Belo Horizonte, Minas Gerais, Brazil; 5 Department of Infectious Disease/Institute of Tropical Medicine, University of São Paulo, Sao Paulo, Brazil; 6 Blood Systems Research Institute, San Francisco, California, United States of America; University Medicine Greifswald, GERMANY

## Abstract

**Background:**

Here, we aimed to gain a comprehensive picture of the HIV-1 diversity in the northeast and southeast part of Brazil. To this end, a high-throughput sequencing-by-synthesis protocol and instrument were used to characterize the near full length (NFLG) and partial HIV-1 proviral genome in 259 HIV-1 infected blood donors at four major blood centers in Brazil: Pro-Sangue foundation (São Paulo state (SP), n 51), Hemominas foundation (Minas Gerais state (MG), n 41), Hemope foundation (Recife state (PE), n 96) and Hemorio blood bank (Rio de Janeiro (RJ), n 70).

**Materials and Methods:**

A total of 259 blood samples were obtained from 195 donors with long-standing infections and 64 donors with a lack of stage information. DNA was extracted from the peripheral blood mononuclear cells (PBMCs) to amplify the HIV-1 NFLGs from five overlapping fragments. The amplicons were molecularly bar-coded, pooled, and sequenced by Illumina paired-end protocol.

**Results:**

Of the 259 samples studied, 208 (80%) NFLGs and 49 (18.8%) partial fragments were *de novo* assembled into contiguous sequences and successfully subtyped. Of these 257 samples, 183 (71.2%) were pure subtypes consisting of clade B (n = 167, 65%), C (n = 10, 3.9%), F1 (n = 4, 1.5%), and D (n = 2, 0.7%). Recombinant viruses were detected in 74 (28.8%) samples and consist of unique BF1 (n = 41, 15.9%), BC (n = 7, 2.7%), BCF1 (n = 4, 1.5%), CF1 and CDK (n = 1, 0.4%, each), CRF70_BF1 (n = 4, 1.5%), CRF71_BF1 (n = 12, 4.7%), and CRF72_BF1 (n = 4, 1.5%). Evidence of dual infection was detected in four patients coinfected with the same subtype (n = 3) and distinct subtype (n = 1).

**Conclusion:**

Based on this work, subtype B appears to be the prevalent subtype followed by a high proportion of intersubtype recombinants that appeared to be arising continually in this country. Our study represents the largest analysis of the viral NFLG ever undertaken worldwide and provides insights into the understanding the genesis of the HIV-1 epidemic in this particular area of South America and informs vaccine design and clinical trials.

## Background

The tremendous genetic variability of HIV-1 is a most prominent feature of the virus and of the HIV/AIDS pandemic. To describe the genetic variation created by mutation and recombination of the viral genome, a unified subtype nomenclature system was established [1]. Of the four principal phylogenetically distinct groups: M, O, N, and P, group M is the predominant circulating HIV-1 group and exhibits the highest degree of genetic diversity. Currently, nine subtypes (A-D, F-H, J,and K), six sub-subtypes (A1–A4, and F1–F2), >70 circulating recombinant forms (CRFs) [http://www.hiv.lanl.gov/content/sequence/HIV/CRFs/CRFs.html] and a number of unique recombinant forms (URFs) of the major group M are recognized [2, 3].

HIV genetic variants are not geographically confined; however, there are circulating clades that are predominant in certain areas [4–6]. For example, in Africa the main reported subtypes are A, C, and D, whereas other countries in Europe, USA, and Australia have reported subtype B as the main clade associated with their epidemic. Subtype C viruses are predominant in South Africa, Ethiopia and India, and CRF01_AE is the major circulating form in Southeast Asia [7–9]. The most prevalent HIV-1 subtypes in the majority of mainland China, Japan's largest neighbor, are B′ (Thailand variant of subtype B; also referred to as Thai-B) and the circulating BC recombinant forms, CRF07_BC, and CRF08_BC, and CRF01_AE [10, 11].

The implications of substantial genetic variation of HIV-1 are extensive including screening, diagnostic testing, disease monitoring, immune response, treatment outcomes, and vaccine design [12]. Therefore, knowledge of genetic variation of HIV-1 is critical for the design of drugs and development of highly anticipated effective vaccines.

In Brazil, the epidemic began in the early 1980s through sexual transmission between men, but now it is primarily spreading through heterosexual transmission. The spread of HIV infection has been complex, characterized by various patterns in different areas of the country [Epidemiological Fact Sheet on HIV/AIDS]. It is estimated that approximately 718,000 people are living with HIV / AIDS in Brazil, representing a prevalence rate of 0.4% of the general population, of which ~ 80% (574,000) have been diagnosed [Coordenãçao Nacional de DST/AIDS]. As in European countries and North America, HIV-1 subtype B is a major genetic clade circulating in Brazil, but the overall prevalence of non-B strains has increased alarmingly, particularly URF BF1 variants[13–15]. Indeed, we have recently reported that at least 38.1% of the 42 sampled group of children and adolescents are infected with HIV-1 BF1 recombinant variants and three of these patients had evidence of dual infections with the same or distinct HIV-1 subtypes [16]. These results shed light on the potential gravity of Brazil's epidemic situation and reinforce the key messages of continual monitoring of the circulating HIV-1 subtypes for directing future vaccine trials.

Investigation of HIV diversity in donor samples can lead to accelerated development and licensing of reliable serologic and nucleic acid amplification assays for donor screening and diagnostic applications. In addition, understanding monitoring the genetic variability of HIV-1 among donors has implications for understanding transmissibility, scrutinizing the prevalence of drug and vaccine escape mutants, and for detecting and monitoring rare variants that may be newly introduced or increasing in the Brazilian donor population. We have recently reported the application of high-throughput near full-length genome (NFLG) and partial human immunodeficiency virus Type 1 (HIV-1) proviral genome massively parallel sequencing [MPS] to characterize HIV in recently infected blood donors at four major blood centers in Brazil [17]. In continuation of our previous study, here, we assessed the genetic variability of HIV-1 on a genome-wide scale in MPS data generated from 257 seropositive blood donors collected between 2007 and 2011 from four Brazilian blood centers of the REDS-II (Retrovirus Epidemiological Donor Study) international program.

## Materials and Methods

### Study population

From 2007 to 2011, 341 HIV+ blood donors from four blood centers were recruited to participate in a case control study to identify risk exposure and motivation to donate. Of these 341 blood donors, 259 blood samples at four major blood centers in Brazil: Pro-Sangue foundation located in São Paulo state (SP, n = 51), Hemominas foundation in Minas Gerais state (MG, n = 41), Hemope foundation in Pernambuco city (PE, n = 96) in the state of Recife, and Hemorio blood bank located in Rio de Janeiro (RJ, n 70) were obtained and submitted for NFLGs amplification and analysis. Of the 259 samples studied, 195 were donors with long-standing infections and 64 donors with lacking stage information. The rationales for collection of these samples and the definition of long-standing infection have been reported previously [18]. All study subjects provided written informed consent. The study was approved by the local ethical review committee of participating institutions,namely, the Pro-Sangue foundation, Hemominas foundation, Hemope foundation and Hemorio blood bank. Also, the study was approved by the REDS-II collaborating centers (Blood Systems Research Institute/University of California at San Francisco, San Francisco, CA) and data-coordinating center (Westat, Inc.) in the United States.

### DNA extraction and amplification of the NFLGs

The genomic DNA used for the PCR analyses was extracted using the QIAamp blood kit (Qiagen) according to the manufacturer’s instructions. The NFLGs from five overlapping fragments were obtained by PCR using the Platinum Taq DNA Polymerase High Fidelity (5 U/μl) (Invitrogen) and were determined by a previously reported method [14, 15]. The amplified DNA fragments from the nested PCR products were separated by gel electrophoresis and purified using Freeze ‘N Squeeze DNA Gel Extraction Spin Columns. Each purified amplicon was quantified using Quant-IT HS reagents (Invitrogen, Life Technologies, Carlsbad, CA), and all five amplicons from a single viral genome were pooled together at equimolar ratios.

### Whole viral genome library preparation

The sequencing library was prepared as described previously [19]. Briefly, one ng of each sample amplicon pool was used in a fragmentation reaction mix using a Nextera XT DNA sample prep kit according to the manufacturer's protocol. Briefly, tagmentation and fragmentation of each pool was simultaneously performed by incubation for 5 min at 55°C followed by incubation in a neutralizing tagment buffer for five min at room temperature. After neutralization of the fragmented DNA, a light 12-cycle PCR was performed with Illumina Ready Mix to add Illumina flowcell adaptors, indexes, and common adapters for subsequent cluster generation and sequencing. An amplified DNA library was then purified using Agencourt AMPure XP beads (Beckman Coulter), which excluded very short library fragments. Following AMPure purification, the quantity of each library was normalized to ensure equally library representation in our pooled samples. Prior to cluster generation, normalized libraries were further quantified by realtime PCR (qPCR) using the SYBR fast Illumina library quantification kit (KAPA Biosystems) following the instructions of the manufacturer. The qPCR was run on the 7500 Fast Real-Time PCR System (Applied Biosystems). The thermocycling conditions consisted of an initial denaturation step at 95°C for five min followed by 35 cycles of [30s at 95°C and 45s at 60°C]. The final libraries were pooled at equimolar concentration and diluted to four nM. To denature the indexed DNA, five μL of the four nM library were mixed with five μL of 0.2 N fresh NaOH and incubated for five min at room temperature. Then, 990 μL of chilled Illumina HT1 buffer was added to the denatured DNA and mixed to make a 20 pM library. After this step, 360 μL of the 20 pM library was multiplexed with six μL of 12.5 pM denatured PhiX control to increase sequence diversity and then mixed with 234 μL of chilled HT1 buffer to make a 12 pM sequenceable library. Finally, 600 μL of the prepared library were loaded on an Illumina MiSeq clamshell style cartridge for paired end 250 sequencing before loading in the cooling section of the MiSeq machine.

### Data analysis

Fastq files were generated by the Illumina MiSeq reporter for downstream analysis and validated to evaluate the distribution of quality scores and to ensure that quality scores do not drastically drop over each read. To take the sequencing error rate into account, we only considered variants detected at a frequency higher than 1% and Phred quality score of >30, i.e., a base call accuracy of 99.9%. Validated fastq files from each viral genome were *de novo* assembled into contiguous sequences and annotated with CLC Genomics Workbench version 5.5 (CLC Bio, Aarhus, Denmark) with default parameters. The contiguous genomic sequence from each virus strain was extracted from the assembly and used for further analysis. Phylogenetic relationships of the newly generated consensus sequences were determined from sequence comparisons with previously published representatives of HIV-1 group M. The full designation of samples, according to WHO-proposed nomenclature, is YYBRCY_XXX, where YY stands for the year of study, BR for Brazil, CY stands for city of enrolment; XXX stands for sample number.

### Screening for recombination events and identification of breakpoints

The *de novo* assembled NFLGs and partial consensus sequences were aligned with reference sequences representing subtypes A–D, F–H, J and K obtained from the Los Alamos database (http://hiv-web.lanl.gov) using MAFFT [20]. Aligned sequences were manually edited and trimmed to the minimal shared length in the BioEdit Sequence Alignment Editor Program. The gap-stripped aligned sequences were screened for the presence of recombination by the bootscan methods implemented in the SIMPLOT program v3.5.1 [21, 22]. The following parameters were used in this method: window size, 350 bp; step size, 30 bp; the F84 model of evolution (Maximum likelihood (ML) as a model to estimate nucleotide substitution; transition\transversion ratio, 2.0; and a bootstrap of 100 trees. In addition, the significant threshold for the bootscan was set at 70%. The jumping profile Hidden Markov Model (jpHMM) [23] was also used to confirm the recombination events and to define the recombination breakpoints according to the HXB2 coordinate system. Recombinant regions of the alignment as determined by the crossover points from the jpHMM and bootscan were analyzed separately by phylogenetic analysis.

### Phylogenetic Analysis

Maximum likelihood phylogenies were constructed using the GTR+I+G substitution model and a BIONJ starting tree implemented in PhyML 3.0 [24]. Heuristic tree searches under the ML optimality criterion were performed using the NNI branch-swapping algorithm. The approximate likelihood ratio test (aLRT) based on a Shimodaira-Hasegawa-like procedure was used as a statistical test to calculate branch support. The maximum composite likelihood in MEGA version 6 [25] was used to calculate the genetic distances between and within sequences. All trees were displayed using MEGA version 6.0 software. Only genuine HIV-1 sequences were included in this analysis.

### GenBank accession numbers

All consensus genome assemblies generated in this study were submitted to NCBI's GenBank database (Accession numbers KT427646-KT427873). The MPS data have also been uploaded to Zenodo: http://dx.doi.org/10.5281/zenodo.46793.

## Results

### Samples

Of the 259 samples used in this study, 195 were from first-time blood donors with long standing/non-recent infection and 64 were donors with unknown stage information. The 259 subjects were identified as non-recent by less-sensitive (LS) or "detuned" enzyme immunoassay [18]. Of note, these isolates were genetically characterized based on *pol* subgenomic fragment from plasma using a conventional bulk sequencing approach and previously published [18]. In the current study, we limited our analysis to the consensus sequences generated from the MPS data. Parallel in-depth analysis of these data including HIV-1 genotypic resistance, tropism, and *quasispecies* are underway.

Of the 259 samples studied, 208 (80%) NFLGs and 49 (18.8%) partial fragments were de novo assembled into contiguous sequences and successfully subtyped. Sequence of the proviral NFLGs and partial consensus sequences revealed that 247 of the generated consensus sequences had open reading frames for most of their genes. The remaining 10 sequences displayed mutations and/or insertions and deletions resulting in frame shifts or premature stop codons.

### HIV variants and sequences

Sequences were obtained for all five overlapped fragments that cover the viral NFLGs of 208 participants (42 from SP, 32 from MG, 81 from PE, and 53 from RJ). Partial sequences were obtained from at least one fragment derived from 49 samples (eight from SP, nine from MG, 15 from PE, and 17 from RJ). Two samples from the non-recently infected group did not amplify for any fragment and were not considered further. The analyses include only the 257 samples whose subtypes were successfully determined. Hypermutated sequences with a predominance of unidirectional G-to-A mutations consistent with APOBEG3G signatures were observed in two samples 010BR_MG005, and 010BR_SP022 and were maintained in the analysis.

### Geographical distribution of HIV-1 subtypes among blood donors in four Brazilian regions

**Fig 1:**
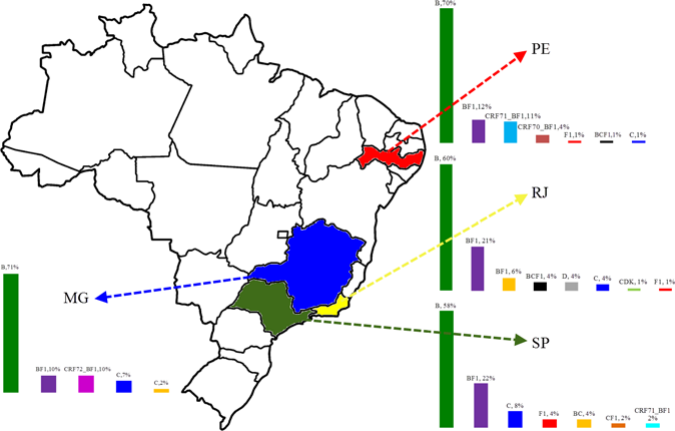
Summary of the subtype distribution of viruses from the 257 long-standing HIV-1 infected blood donors analyzed. The number of viruses that belong to each subtype is indicated in the relevant bar chart section. The regions of origin are color-coded and indicated by arrows.

A total of NINE different unique HIV subtypes and three different BF1 CRF variants were identified through NFLGs and partial genome analysis. Fig 1 shows the regional distributions of HIV-1 subtypes and illustrates the substantial HIV genetic diversity in Brazil. In particular, there are at least five subtypes circulating in MG, seven in PE, seven in SP, and eight in RJ. The genetic subtype distribution did not differ significantly among the four regions. Approximately 82.2% (74 of 90) of non-subtype B strains in this study were identified as recombinant variants, representing 28.8% (74 of 257) of the total successfully sequenced samples. Subtype B is predominant in the four regions (167 of 257 [65%]); the lowest prevalence was observed in SP (29 of 50 [58%]) and RJ (42 of 70 [60%]), and the highest prevalence was seen in MG (29 of 41 [71%]). BF1 recombinants including the newly described CRF 70 and 71 BF1 [26] were identified in 27% (26 of 96) of subjects from PE, 21.4% (15 of 70) from RJ, 24% (12 of 50) from SP, and 19.5% (8 of 41) subjects from MG. Subclade F1 was observed at low prevalence in SP, PE, and RJ, where it represents 4% (2 of 50), 1% (1 of 96), and 1.4% (1 of 70) of the circulating strains, respectively. Subtype C was also seen at lower prevalence in all regions, 8% in SP. 7.4% in MG, 2.8% in RJ and 1% in PE (Fig 1). Two sequences belong to subtype D were detected in RJ. A detailed description of the NFLG of these two sequences has already been published elsewhere [26].

### Characterization and distribution of non-recombinant HIV-1 NFLG subtypes

**Fig 2:**
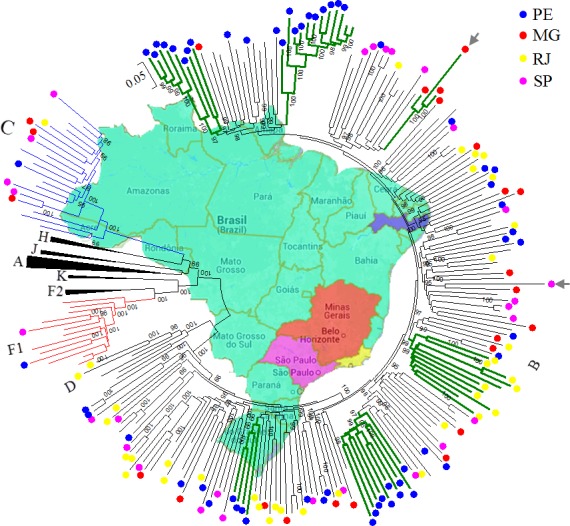
Phylogenetic tree constructed using a ML method from the non-recombinant NFLG sequences of 149 blood donor samples, 53 non-redundant B, eight subclade F1, and eight subtype C sequences from Brazil retrieved from GenBank database. Besides these sequences, the tree also included 37 reference strains (Los Alamos database) representing subtypes A-D, F-H, J and K. Sequences identified in this study are indicated by circles and the geographic origin of each sequence is color-coded. Monophyletic clusters from the same geographic origin are indicated by thick green branches. For clarity purposes, the tree was midpoint rooted. Gray arrows indicate hypermutated sequences with a predominance of unidirectional G-to-A mutations (010BR_MG005, and 010BR_SP022). The aLRT values of at least 80% are indicated at nodes. The scale bar represents 0.05 nucleotide substitutions per site.

The phylogenetic analysis of the 208 HIV-1 incident samples with NFLG sequences revealed 149 non-recombinant variants (71.6%) and 59 recombinants subtypes (28.4%) over a mean length of 8536 bp (range: 8142–9298). Fig 2 shows the ML tree of the 255 NFLG sequences, including the 149 non-recombinant sequences generated in this study, 53 non-redundant B, eight subclade F1, and eight subtype C sequences from Brazil retrieved from GenBank database. Besides these sequences, the tree also included 37 reference strains (Los Alamos database) representing subtypes A-D, F-H, J, and K. The 136 genuine subtypes B from this study displayed an overall average distance of 6.4% and were seen in the four regions; 57 in PE, 22 in MG, 23 in SP, and 34 in RJ. Two sequences from RJ tightly clustered with subtype D reference sequences. The genetic distance at the nucleotide level between these two sequences and other isolates within subtype D reference sequences was 10.7%. As depicted in Fig 2, subtype C NFLG was detected in all regions; one in PE, three in MG, four in SP, and one in RJ. One subclade F1 was detected in SP and PE and both clustered on a sub-branch intrinsic to subclade F1 from Brazil. Close inspection of the ML tree indicated potentially significant clustering of six groups composed of three to 10 infections caused by subtype B with significant aLRT support values (an excess of 95%) and mean intracluster genetic distance ranging from 5.3% to 11.2%. Four clusters were limited to blood donors from PE and two other clusters were limited to subjects from MG and RJ indicating a potential epidemiological link because of a common source of infection or existence of a larger HIV-1 transmission cluster.

### Characterization and distribution of the NFLG recombinant sequences

**Fig 3:**
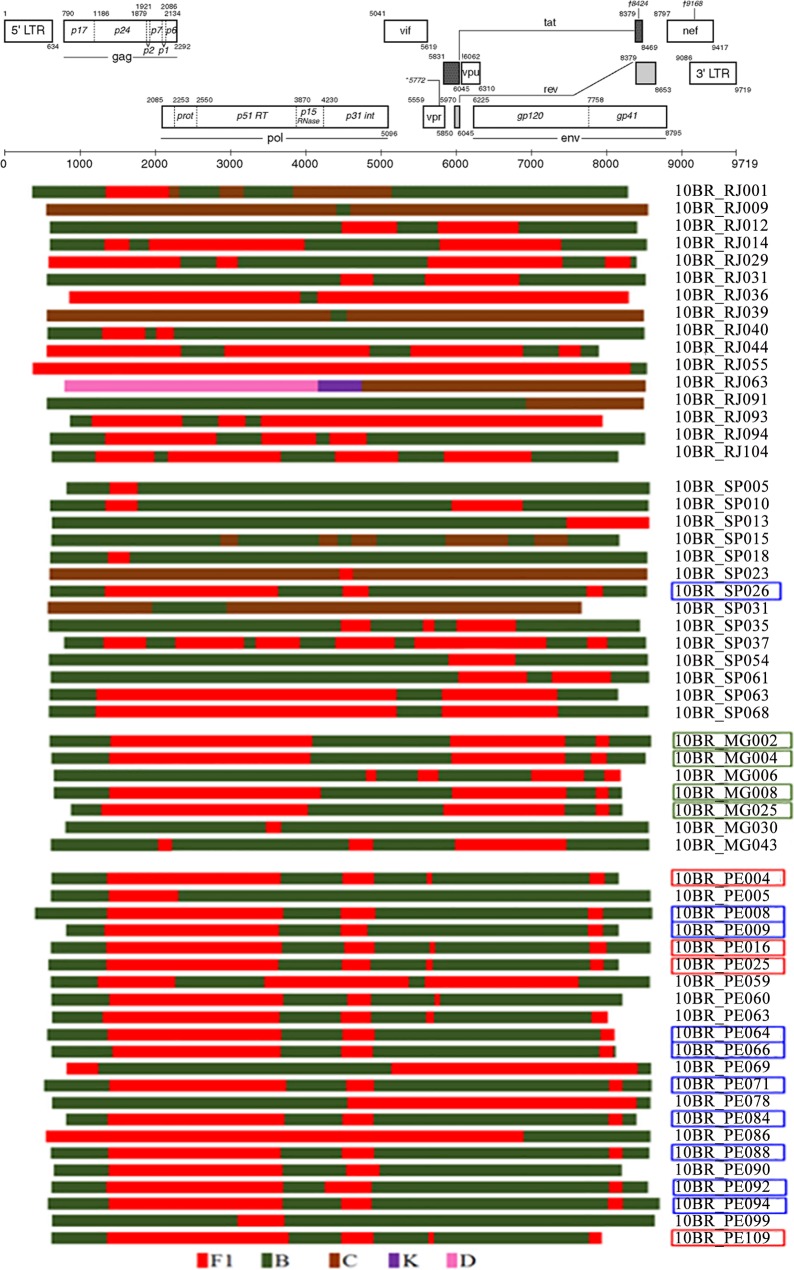
Schematic representation of the NFLGs and breakpoint profiles of the recombinant sequences from proviral DNA are generated by a deep sequencing approach identified in this study. Sequences were mapped relative to the HXB2 numbering system. HIV subtypes are color codes and indicated at the bottom. CRF70_BF1 variants are boxed with red rectangle squares, CRF71_BF1 with blue rectangle squares, and CRF72_BF1 with orange rectangle squares.

Among the 59 NFLG isolates with strong evidence of recombination, 51 (86.4%) were unique mosaic isolates consisting of subtype B and F1 (n = 32), CRF70_BF1 (n = 4), CRF71_BF1 (n = 11), and CRF72_BF1 (n = 4). The remaining eight sequences belonged to four different HIV-1 recombinant subtypes and all contained fragments of subtype C in their genomes (Fig 3). The genetic heterogeneity is remarkable in RJ where at least four different patterns of recombination were observed. As shown in Fig 3, two distinct BF1 recombinant profiles, with four samples in profile I and nine in profile II were detected in samples from PE. These sequences were registered with the Los Alamos National Database as CRF70_BF1 (red rectangle box) and CRF71_BF1 (blue rectangle box). Detailed information of these sequences has recently been published by our group [26]. One BF1 sequence from SP (10BR_SP026) displayed a recombinant structure identical to the newly described CRF71_BF1. Recombination analyses of sequences from MG show four sequences with identical recombinant profile essentially consisted of subclades F1 and subtype B as parental sequences (green rectangle box, Fig 3) and appeared different from all previously documented CRFs in Brazil and South America. These sequences have recently been identified as CRF72_BF1 [26]. According to our estimate, the new CRF72_BF1 accounts for 9.7% (4/41) of the HIV-1 circulating strains among blood donors in MG and is responsible for more than 44% (4/9) of infections caused by all recombinant variants circulating in that region. To further identify more HIV-1 sequences with a similar recombinant structure, all NFLGs URFs and CRFs of BF1, BC, CF1, and BCF1 sequences were retrieved from Los Alamos database and analyzed together with our unique recombinant NFLG sequences. The results revealed no evidence of a relationship with any previously reported variants and thus these sequences are representing novel HIV-1 unique recombinants.

### Characterization and distribution of HIV-1 subtypes by partial sequencing

**Fig 4:**
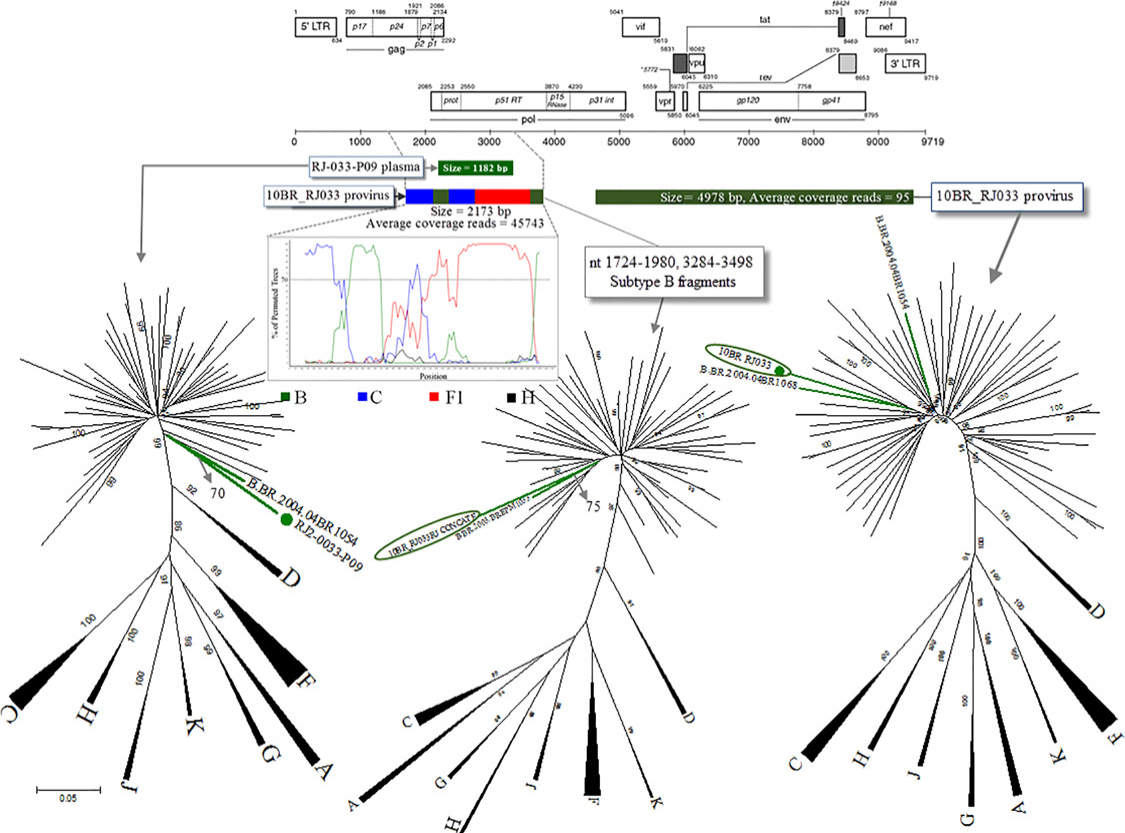
Phylogenetic trees were constructed using a maximum-likelihood method from partial *pol* plasma RNA virus (1182 bp), concatenated regions of subtype B sequences (provirus nt 1724–1980, 3284–3498) and proviral DNA (4978 bp fragment) from patient 010BR_RJ033 along with 53 non-redundant Brazilian subtype B and HIV-1 reference sequences from the Los Alamos HIV-1 database representing 11 genetic subtypes. The recombinant proviral fragment (2173 bp) is evidenced by a Bootscan plot. Average coverage reads of both proviral fragments are indicated.

Partial sequences were obtained from at least one fragment derived from 49 blood samples. Among these sequences, 31 (63.3%) were pure subtype B, three (6.1%) were pure non-B subtypes, and 15 (30.6%) were recombinant strains including one CRF71_BF1 from SP. A high prevalence of recombinant variants was detected in seven of 17 (41.2%) subjects from RJ. In one donor (10BR_RJ033), the amplification and sequencing of proviral DNA were successful in a recombinant CBF1 fragment I (nucleotide position from start of HXB2 genome 1311–3498) and a non-recombinant subtype B fragment II (nucleotide position from start of HXB2 genome 4605–9532) as shown in Fig 4. Fragment I yielded 497,763 sequencing reads after filtering with a median coverage of more than 45x10^4^ fold while fragment II (4978bp) yielded 3364 of MPS reads with a median coverage read depth of >90 fold. Further analysis was performed to determine whether the two proviral fragments were derived from the same virus. For this purpose, the phylogenetic clustering profile of the non-overlapped subtype B fragments were compared to a number of additional Brazilin subtype B and other HIV-1 reference sequences to increase our confidence in the analyses and provide a broader perspective. As indicated in Fig 4, the 10BR_RJ033 placed the concatenated subtype B fragment (nt 1724–1980, 3284–3498) with BBR2003_BREPM1033 (GenBank: EF637050) (aLRT 75%), while fragment II strongly (aLRT 95%) grouped with BBR2004_04BR1068 sequence. The clustering profiles to subtype B references were significantly different from both fragments in this patient indicating recombination or infection with distinct subtype B variants.

### Comparison of HIV-1 subtypes in plasma and PBMC

The relationships of the proviral DNA sequences from patients’ PBMCs to the RNA sequences derived from plasma viruses within the same regions (n = 230) were calculated for each patient to assess the viral diversity in both compartments. Of these 230 samples, 180 were derived from non-recently infected donors. Surprisingly, the intraindividual plasma and proviral sequence variation in four (2.2%) non-recently infected blood donors (10BR_MG005, 10BR_SP022, 10BR_RJ024, and 10BR_RJ033) in the partial *pol* regions were remarkably high (≥ 6%), indicating that the plasma viruses were derived from a population significantly distinct from those of the cellular sources, results consistent with dual infection with different subtypes. Again, multiple infections with distinct viruses of subtype B and CBF1 recombinant in the same genomic region were observed in patient 10BR_RJ033 in plasma and PBMCs, respectively (Fig 4). The clustering profiles of RNA sequence and proviral fragment II to subtype B references were significantly different in this patient indicating either recombination or coinfection with distinct subtype B variants. Thus, the overall analysis indicates that the infection in this donor is founded by two or three genetic lineages.

## Discussion

In this study, we report the high degree of genetic variability of HIV-1 using nearly full genome sequencing and partial genomic fragments of proviral DNA of samples from blood donors from four major blood centers in Brazil. The results presented confirmed that subtype B is still the main HIV-1 variant in Brazil, as reported previously for the same sample of subjects from REDS II [18], and are concordant with data from other Brazilian studies [15, 27, 28]. However, this study found an additional eight cocirculating subtypes, three CRFs, and 20.6% novel unrelated URFs. A high intersubtype diversity (28.9%) observed in this study is consistent with the diversity reported in our previous study [17] and is even higher than what has been reported in previous studies, although results were difficult to compare, because our study was based on NFLGs and larger fragments whereas previous studies in Brazil used short partial sequences [18, 29, 30]. The high prevalence of HIV-1 inter-subtype recombinant viruses in this group of blood donors is likely to reflect the presence of highly exposed individuals and social networks of HIV-1 transmissions in Brazil. Also, analysis of NFLGs and larger fragments allows the detection of more recombinant viruses. Except of CRF70, 71 _BF1and from PE and CRF72_BF1 from MG, most of the mosaic viruses described in this study were novel variants with diverse profiles of intersubtype recombination and none had a similar structure. This could indicate that these URFs are evolutionary relics [31] or that none of these variants has a selection advantage in the population. Additional studies are needed to define the replicative kinetics and transmissibility in order to better understand the evolutionary pathway of these URFs.

Of note, most of the recombinant viruses identified in this study contained the co-circulating HIV-1 subtypes B, C, and/or F1 in Brazil in their genomes. Based on our analysis, some of these recombinant variants were particularly complex mosaics containing various recombination crossover sites among three HIV-1 subtypes. Inter-subtype recombination events are generally the result of super-infection or coinfection with viruses of more than one subtype and most frequently detected in high risk populations with heavy exposure to multiple subtypes [32, 33].

Little regional differences were observed in subtype distribution, but in each region, at least five to eight different clades co-circulated. Overall, subtype B was predominant in MG and PE, with decreasing proportions in RJ and SP. The largest variety of HIV strains was found in RJ with a total of eight subtypes and 40% of non-B clades. Of note, RJ is the third largest metropolitan area in Brazil and is considered one of the main tourist destinations in the Southern Hemisphere, receiving people from other South American countries and all over the world, which could have implications about fueling the epidemic in this city through superinfection and recombination.

The strategy used in this study also permitted the description of mixed infections with distinct subtypes. In four samples with mixed infections, one appeared to harbor three different subtypes and that variant seen in the plasma was completely absent in the PBMCs, a result suggesting differential sources of infecting viruses. It is also conceivable that the discordances in the HIV subtypes in PBMCs and plasma are due to low-level minority strains present, which are not detected with bulk plasma sequencing or that the replicating viruses shed in the plasma were more fit. Simultaneous infections by the same HIV subtype with distinct variants or by different HIV subtypes have been well documented [16, 34–38] and will continue to enable the genetic diversity and emergence of new virus strains. It is unclear from this study whether the occurrence of multiple distinct HIV strains was the result of superinfection with a second variant at a later time or whether simultaneous infection with multiple viral strains occurred during a single transmission event. However, the circulation of multiple subtypes in Brazil supports the possibility of both scenarios. The overall results indicate that the rate of HIV-1 mixed infections within this Brazilian group of non-recently infected donors is higher than 2%. This estimate is lower than the rate of 12% that was recently observed by our group in 45 recently infected first-time blood donors at the same four blood centers in Brazil [17]. The low prevalence of mixed infection in our long-standing HIV-infected blood donors could indicate a protective mechanism at work, such as immune responses that evolve over time or the ability of the original virus to ward off acquisition of another.

Given the extensive genetic variability observed among the low risk blood donors, it may be suspected that this diversity is much more complicated among other high-risk groups with a tight transmission chain. Therefore, there is a strong need for regular and systematic surveillance of HIV-1 in Brazil. The high number of HIV-1 subtypes co-circulating and diverse recombinant viruses observed in our study is consistent with an old mature epidemic in Brazil and represents a real challenge for future vaccine development, as well as for efficiency of antiretroviral treatment and diagnostic tests.
